# Smoking Induced Hemolysis: Spectral and microscopic investigations

**DOI:** 10.1038/srep21095

**Published:** 2016-02-19

**Authors:** Vadivel Masilamani, Khalid AlZahrani, Sandhanasamy Devanesan, Hadi AlQahtani, Mohamad Saleh AlSalhi

**Affiliations:** 1 Department of Physics and Astronomy, King Saud University, Riyadh, KSA- 11451, Saudi Arabia; 2 Research Chair in Laser Diagnosis of Cancers, College of Sceince, King Saud University, Riyadh, KSA- 11451, Saudi Arabia; 3 King Abdullah Institute for Nanotechnology, King Saud University, Riyadh, KSA- 11451, Saudi Arabia.

## Abstract

Smoking is one of the major causes of lifestyle associated mortality and morbidity such as cancer of the oral cavity and lungs, and also cardiovascular diseases. In this study, we have provided evidences for the smoking-induced hemolysis using two methods: spectra of blood components and atomic force microscopic analysis of surface morphology. A total of 62 subjects (control = 31; smoker = 31: 21 male; 10 female in each set) were considered for the study. The findings indicate that smoking leads to potholes on the surface, swelling of shape, rupturing of erythrocytes, removal of hematoporphyrin and flushing into the plasma as metabolites of the erythrocyte. The overall morphology of the erythrocytes of the smoker group appears more like a Mexican hat. The mean surface roughness was 5.5 ± 3 nm for the smoker group, but 1.2 ± 0.2 nm for the control group. Such damages might help the toxins, (CO, peroxidants, aldehydes etc.,) to gain easy access and get strongly absorbed by the hemoglobin, leading to enhanced rates of hemolysis as shown by the spectral features of metabolites. This indicates that the average life span of the smoker’s erythrocytes is significantly less than that of the control group.

Tobacco smoke contains at least 3500 chemicals such as carcinogens, mutagens, free radicals, heavy metals, and even radioactive materials^1^,^2^. According to the American Cancer Society’s report, smoking accounts for 30% of all cancer deaths in the US; further, 80% of lung cancer deaths are exclusively due to smoking^3^. In addition, smokers are at three-times greater risk of cardiovascular diseases than the nonsmokers. Moreover, smoking causes more deaths than the combined mortality due to HIV and motor vehicle injuries^2^,^3^. In addition, smoking causes chronic nonfatal diseases such as cataract, arthritis, erectile dysfunction, etc. However, only a few studies have investigated smoking-induced damage to the blood^4^,^5^,^6^,^7^,^8^.

Optical biopsy is a new technique where the light of UV or visible radiation is employed as a tool to probe the intrinsic conditions of tissues (normal, benign, or malignant) or body fluids (blood, urine, saliva, etc.). When the light of a particular wavelength falls on a tissue or body fluid, it undergoes scattering or absorption because light photons interact essentially with the biomolecules. Some of these biomolecules can also produce fluorescence or incoherent molecular scattering leading to Raman shifts. Therefore, the different spectra obtained from such biomolecules serve as biomarkers of different diseases. Many studies have focused on the fluorescence and Raman spectra of a variety of malignant tissues *in vivo* and *in vitro*^9^,^10^. Similar studies have focused on the detection of malignancy of lung, liver, etc.^11^,^12^ (and inherited blood disorders, such as thalassemia and sickle cell anemia)^13^ from the spectral features of blood and urine^14^. The fluorescent spectral technique can be used for molecular diagnosis, and the present study can be considered as a logical extension of the spectral technique of blood components for monitoring smoking-induced damage on erythrocytes.

The atomic force microscopic (AFM) analysis is the morphological and structural analysis of erythrocyte membrane to confirm the finding of spectral investigations. The AFM is a scanning probe microscopy (SPM), with the resolution of the order of a few nanometers and 1000 times better than the optical diffraction limit. By using an AFM, it is possible to measure the roughness and hardness of a sample surface at a high resolution. In comparison to the scanning electron microscope (SEM), AFM can provide a three-dimensional surface profile. In short, AFM is one of the cutting-edge techniques for imaging and measuring at micro and nanometer scale^15^,^16^,^17^.

## Results

### Spectral Analysis

**Figure 1:**
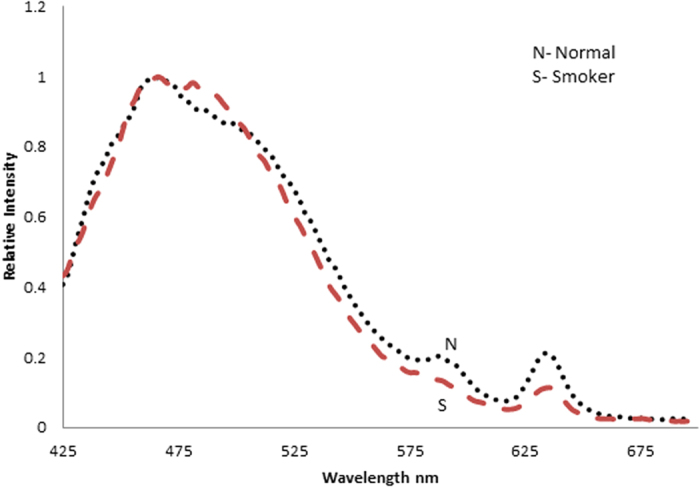
Fluorescence emission spectra (FES) of acetone extract of RBC (excitation at 400 nm). N - Control sample, S- Smoker sample.

In this report, the typical FES of acetone extract of erythrocyte of the control (normal) and heavy smokers are presented; both were excited at 400 nm. Figure 1(a) gives the typical FES for the control; it has a broad band at 470 nm due to the fluorescence of NADH (found in the acetone extract of residual plasma). The next bands at 585 nm and 630 nm are due to the basic and neutral form of protoporphyrin, an essential component of hemoglobin of erythrocyte, respectively. In fluorescence spectroscopy, it is conventional to normalize the spectra and then measure the relative intensities of different peaks so as to reduce inter-instrumental errors.

From that point of view, a ratio R_1_ = I_630_/ I_585_ (I stands for intensities at 630 and 585 nm) were measured for the control set, which gave R_1_ = 1.15 ± 0.1(p < 0.05). A similar measurement for the smoker set gave R_1_ = 0. 76 ± 0.2 (p < 0.05). This means the porphyrin contents in the erythrocytes of the smoker group was only about 66% of that of the control group.

**Figure 2:**
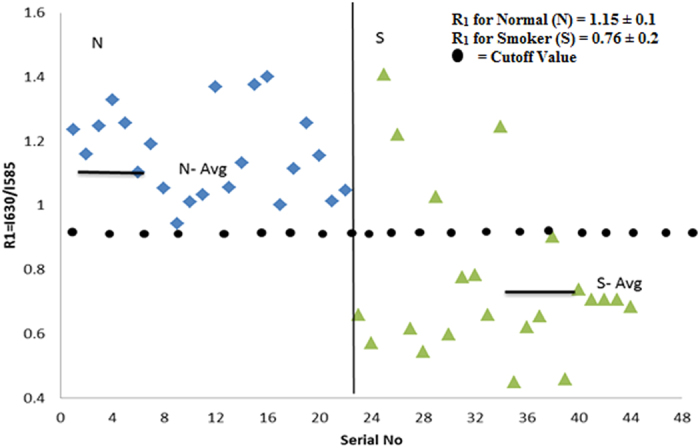
Distribution of R1 = I_630_/I_585_ ratios, as obtained from Fig. 1. N- Control sample, S- Smoker sample.

Figure 2 gives a representation of scatter plot of ratio parameters R_1_ (a measure of porphyrin concentration in the erythrocyte, as shown in)^10^,^11^,^12^,^13^,^14^ for the control and smoker sets. It was observed that most of the R_1_ values for the smoker group were less than the cutoff value, R_1_ = 1 (arbitrarily chosen).

**Figure 3:**
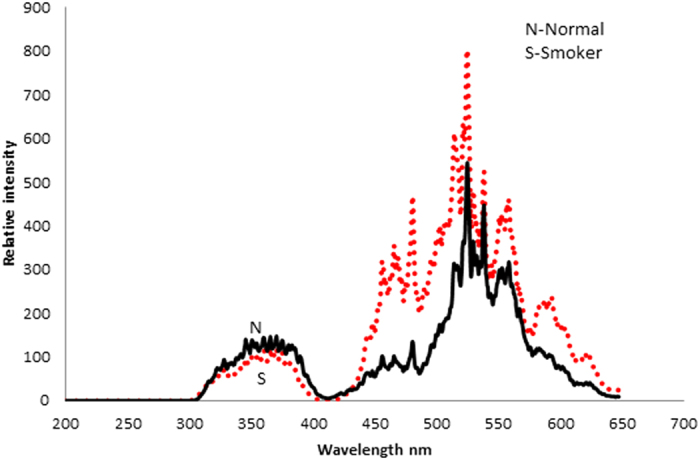
Synchronous emission spectra (SES), of blood plasma of N- Control sample, S- Smoker sample.

Figure 3(a,b) show the typical SES of blood plasma of the control and smoker groups respectively. Here, the peak at 360 nm is due to the amino acid tryptophan, the peak at 460 nm is due to the enzyme NADH, and the peak at 525 nm is due to the metabolite FAD. The peaks at 585 nm and 630 nm are due to porphyrin metabolites. In order to highlight the contrast between the smoker and the control groups, normalization has been done with reference to the peak at 360 nm. It can be seen that R_2_ = I_525_/I_360_ = 2.6 ± 0.2 and R_3_ = I_450_/I_360_ = 0.63 ± 0.1 2 for the control group. However, R_2_ = 4.0 ± 0.6 and R_3_ = 1.80 ± 0.75 for the smoker group. This indicates that, in the plasma of the smokers, the metabolites such as NADH, FAD and hematoporphyrin (with unresolvable bands at 585 and 620 nm) are about twice in concentration as compared to that of the control group. In other words, smoking has caused the rupture of hemoglobin in the erythrocytes to flush it into the plasma.

**Figure 4:**
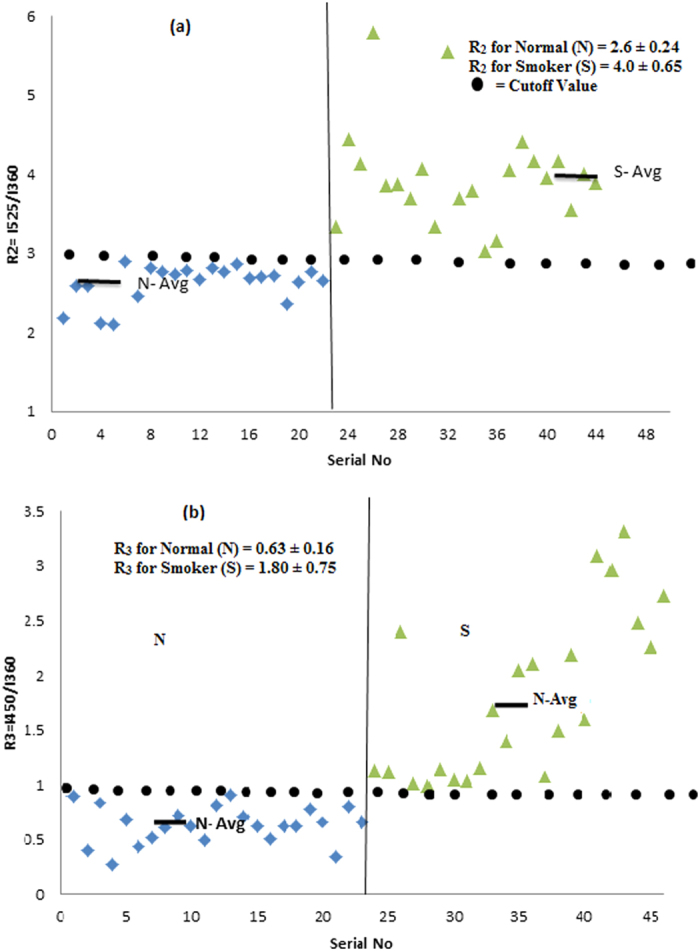
(**a**) Distribution of R_2_ = I_525_/I_360_ ratios, as obtained from Fig. 3. N- Control sample, S- Smoker sample. (**b**) Distribution of R_3_ = I_450_/I_360_ ratios, as obtained from Fig. 3. N- Control sample, S- Smoker sample.

Figure 4(a) gives the scatter plot of ratio parameters R_2_ (a measure of FAD concentration in the plasma of blood) for the above two sets. Figure 4(b) provides the scatter plot of R_3_ (a measure of NADH, in blood plasma) for above the two sets. All the above set of figures, based on the fluorescence spectral features of erythrocyte and plasma, strongly indicates that smoking leads to premature hemolysis.

**Figure 5:**
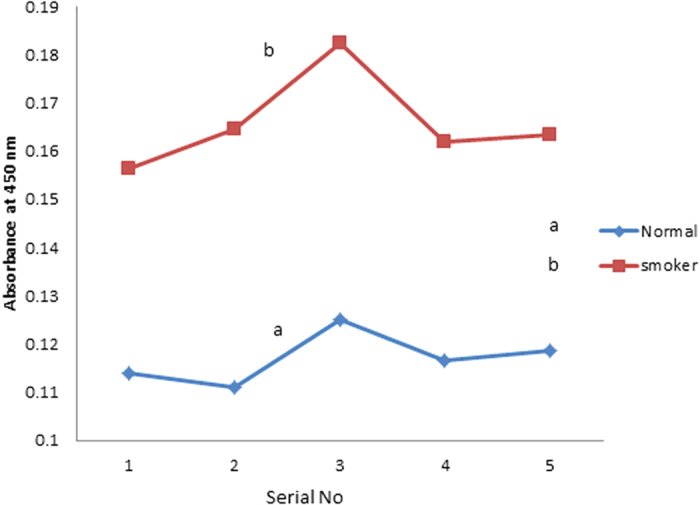
NADH metabolite concentration as measured by ELISA. N- Control sample, S- Smoker sample.

Figure 5 gives the NADH level for plasma of a few randomly chosen smoker and control (N = 5) as measured by ELISA technique. The NADH for smokers was 40–50% elevated than normal controls. A very similar results have been observed for NADH dehydrogenase subunit (2 237 Leu/Met) enhancement for Japanese smokers^18^.

### AFM analysis

**Figure 6:**
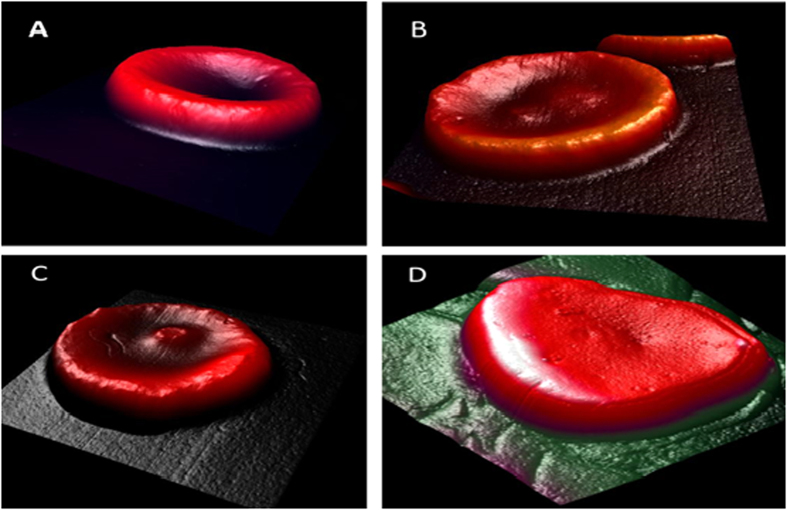
Transformation of RBC shape due to cigarette smoking. (**A**) AFM 3D-image 12 × 12 μm of healthy non-smoker RBC. AFM 3D- images 12 × 12 μm for deformation of RBCs due to heavy smoking for long time.

A representative AFM image of the RBCs from the control group is shown in Fig. 6(A). The images showed that most of the RBCs from the healthy, non-smoking individuals (control) have a typical discoid shape. In contrast, at least 60% of the scanned cells from the smokers group showed RBCs to be remarkably different from the typical discoid shape. Three examples of the distortion of RBCs due to smoking are illustrated in Fig. 6(B–D). The overall morphology exhibited serious deformities, particularly, at the center. The cell surface architecture was massively deformed with the loss of the characteristic biconcave shape of the RBCs. The center of the cell was swollen at different areas with hump-like structures, which were similar to English or Mexican hats. In addition, the edges of the cell had also become irregular in shape.

Recent studies have reported similar changes in the shape of RBCs of the smokers group using the SEM^15^,^16^,^17^. Under abnormal physiological conditions, various endogenous or exogenous factors may transform the shape of RBCs, affecting their ability to function.

**Figure 7:**
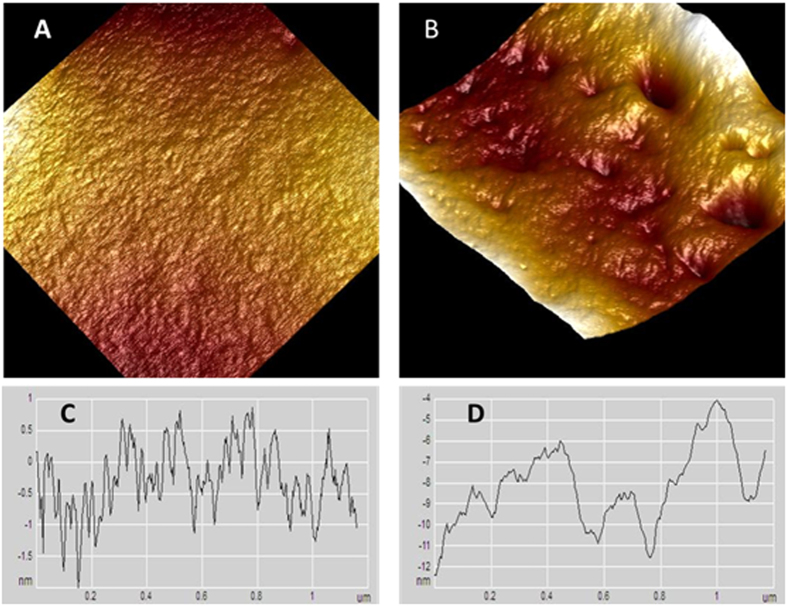
Nanostructure of RBC cell membrane from healthy non-smoking individual (**A**) and long term smoker (**B**). Profiles of membrane nanostructure for non-smoker (**C**) and smoker (**D**).

The nanostructure of RBCs was also investigated by AFM. The high magnification of RBCs of the control group showed smooth cell membrane nanostructure without significant irregularities (Fig. 7(A)). In contrast, serious damages were found in the nanostructure of the RBCs of the smoker group (Fig. 7(B)). Compared to the RBCs of the control group, most parts of the membrane surface appeared uneven, with fissures and crater-like structures. Such morphological changes of cell membranes were observed in many, but not all, RBC samples from the smokers group. The structure of the cell membrane was found to have extensive potholes and eruptions, which we may be termed as nano rupture and nano hemolysis.

**Figure 8:**
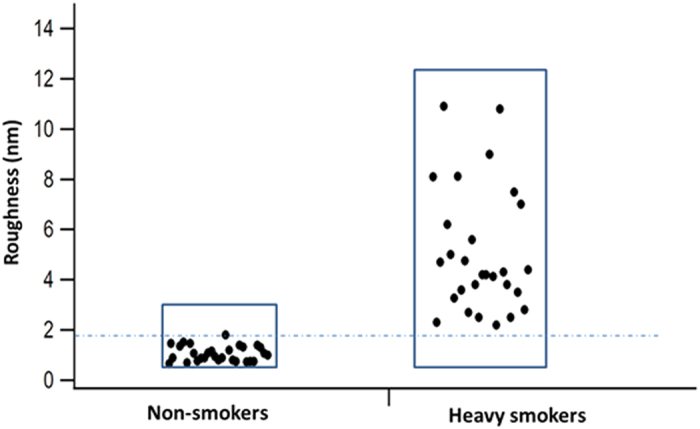
RBC membrane roughness in the control cells (non-smoker) and smokers.

Figure 8 illustrates a scatter plot of the roughness of the cell membrane of RBCs from control and smokers taken randomly at different positions across the cells. The roughness was measured at different positions on the RBCs’ cell surfaces, and the area scanned was less than 1 μm^2^ for each measurement.

The roughness of the cell membranes of the RBCs for the control was 1.2 ± 0.2 nm, but for the long-term smokers, it was in the range of 5.5 ± 3.1 nm, which is approximately four times higher than that of the control cells. The change of cell membranes’ structures and the increase in roughness are significant and indicative of profound alterations of the cell membrane ultrastructure.

**Figure 9:**
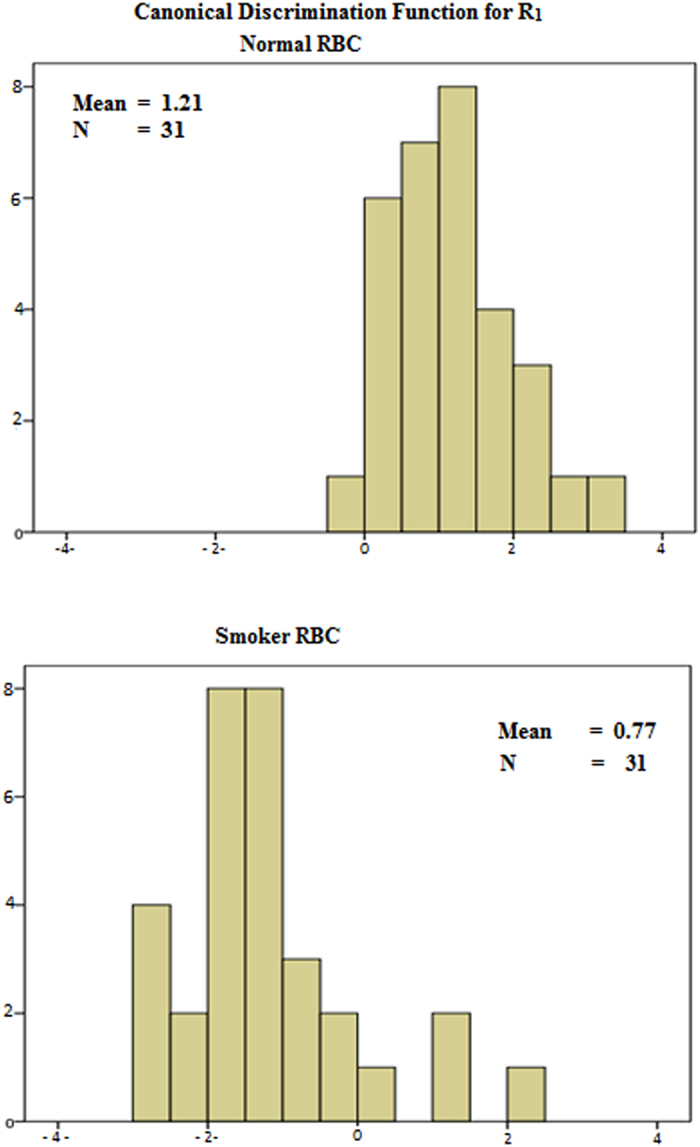
Canaonical Discriminant for spectral parameters R1 (of normal control and smoker).

In order to ensure statistical significance in classifications of the two sets (control and smokers) canaonical discriminant analyses were done for all the ratio parameters and only the essential features of a few are shown in Fig. 9 and Table 1.

## Discussion

Though extensive work has been done on the impact of smoking on the onset of malignant cell transformation and cardiovascular diseases, only scanty reports are available on the impact of smoking on erythrocytes^19^,^20^.

The experimental evidences from the two independent techniques presented here strongly indicate that smoking damages the cell membranes and intracellular content of the RBCs. The above damage mechanism can be presented as follows: The particulate matter of micro and nanometer size, heavy metals, radioactive materials, free radicals and peroxidant present in the inhaled cigarette smoke damage the surface of the cell membrane by pitting and etching. This is similar to the meteorites hitting the lunar/geo surface. The most probable targets are proteins such as collagen, elastins and lipids such as polyunsaturated fatty acids^21^ that are found rich on the surface of erythrocytes. Such surface damages cause toxins such as benzene, carbon monoxide, hydrogen peroxide etc., to enter into the erythrocyte cell membrane^21^. It is important to draw attention that most of the erythrocytes might be exposed to such harsh treatment over and over again in its life span of 120 days producing a cumulative surface damage. Since hemoglobin has 200 folds greater affinity for CO, carboxy hemoglobin is produced copiously^22^ that eventually ruptures the hematoporphyrin from the hemoglobin and flushes into the plasma stream. In other words, the normal lifespan of 120 days of RBCs is reduced to 80–85 days because of smoking. It is difficult at this stage of the investigation to be more quantitative or accurate than this.

It may be worthwhile to compare the spectral features of blood components of patients with thalassemia and sickle cell diseases. In these inherited blood disorders, the concentration of metabolites were six or eight folds higher compared to that of the control group^15^,^16^,^17^. In other words, the reduced life span of erythrocytes is due to the inherited blood disorders in the case of thalassemia but acquired lifestyle in the case of smoking.

Another noteworthy point is the bloated hat-like structure of the erythrocytes shape is due to the unusual chemical reaction initiated by a host of toxins such as CO and benzene. The evolution of certain abnormal gasses inside the erythrocytes leads to abnormal shape, filling the discoid shape of the normal cell. A very similar observation has been made by a study of mechanical properties of RBC. This report has shown evidences for loss of resilience due to enhanced shear and bending moduli of RBC on exposure to free radicals and hyperoxides^21^. This could result in the loss of structural elasticity of the erythrocytes and retard the easy movements in the blood vesicles of heart and penis. In addition, the nano rupture and hemolysis might deposit crusts of metabolites on the walls with the eventual reduction in oxygen transport, resulting in cardiac arrest^23^ and erectile dysfunction.

## Materials and Methods

A total of 62 subjects were considered for the study. Of them, 21 were male smokers and 10 were female smokers (of 10–15 pack year with median dose of 12) aged 25–50 years with a median age of 35. who were grouped in the smokers group. The remaining 31 were normal subjects who were carefully selected (median age 35) for age and sex (control group 21 male and 10 female). All the volunteers in this study were regular employees of the King Saud University and the King Khalid University Hospital (KKUH). The protocols of the study were explained to the subjects and their written informed consents were obtained; also none had any specific disease as per the written declaration. The permission for this study was obtained from the institutional review board (IRB), of KKUH. The study was carried out in the Laser Diagnosis of Cancer Research Chair and according to the guidelines of Ministry of Higher Education, King Saud University, College of Medicine and King Khalid University Hospitals in Saudi Arabia, with the approval of ethical committee letter [E-12-754].

Intravenous blood (5 ml) was drawn from each subject and collected in an EDTA vial, which contained the anticoagulant coated on the inner wall. Each tube was rocked for five times gently for even mixing, followed by centrifugation (3000 RPM, 15 min) for each sample to separate the cellular components from the plasma. Then about 1 ml of supernatant plasma, a greenish-yellow liquid, was pipetted out and drawn into a sterile glass tube (sample 1). From the EDTA vial, the top buffy coat was carefully pipetted and discarded; the residual, thick jelly-like cellular components contained mostly erythrocytes. Then 0.5 ml of this residue was again pipetted out, washed with saline water and centrifuged (3000 RPM, 10 min). The residue, which contained mostly erythrocytes without any impurities, was taken for AFM (sample 2). Another 0.5 ml of erythrocytes was lysed with 1.5 ml of spectroscopic grade acetone. The mixture was shaken well for efficient extraction of biomolecules of the erythrocyte. This was centrifuged (3000 RPM, 15 min), and the clear supernatant, containing mostly fluorescent biomolecules, was taken for spectral analysis (sample 3).

When a biomolecule absorbs a photon, it gets excited and remains in the excited state for a few nanoseconds and re-emits as fluorescence, which acts as a fingerprint for a particular molecule and their environment. Out of a large number of such bio-molecules such as proteins or amino acids, only a dozen of them fluorescence in the range of 200 to 800 nm. The relative proportion of such fluorescent molecules acts as biomarkers of a certain set of well-defined diseases^9^,^10^,^11^,^12^,^13^,^14^.

In a spectrofluorometer such as P E LS 55 (Perkin Elemer LS 55, USA) that was used in this study, there are two gratings for emission, excitation or synchronous scans. When the excitation grating is fixed and allowed to select a light of particular wavelength (say 400 nm) and is used to excite the sample (say acetone extract of erythrocyte), the emission grating is scanned from 425 nm to 700 nm to map the fluorescence profile of a set of biomolecules in that range. This is called fluorescence emission spectra (FES). On the other hand, when the excitation and emission grating are set at 10 nm offset and rotated synchronously, one obtains a synchronous emission spectra (SES) of a host of molecules. These molecules include nicotinamide adenine dinucleotide (NADH), flavin adenine dinucleotide (FAD), porphyrin, etc., which have partially overlapping emission profiles in complex systems such as the blood plasma. It is important to emphasize that FES and SES are variants of fluorescence spectroscopy^9^,^10^,^11^ and that both spectra can be considered to be optical analogues of X-ray radiography and computed tomography (CT) scan^10^,^11^,^12^,^13^,^14^.

In order to confirm the spectral data of hemolytic end products, one of the fluorescent metabolite, NADH, was measured by the conventional biochemical analysis using ELISA [(HT Bio-Tech USA) with the ab65348 kit obtained from ABcam UK. In brief, 5 μL of plasma of smoker and 45 μL of NADH extraction buffer and 100 μL reaction mix were put into each well of ELISA. An additional 10 μL NADH developer was then added and incubated for 2–3 hours at room temperature and absorbance (OD) at 450 nm was measured to quantify NADH level in each plasma sample. Such measurement was done in four wells and the reported value for each sample is the average absorbance obtained from four wells. Similar producers were done for the control samples too.

A droplet of the erythrocytes was spread on clean round glass cover slips of 12 mm diameter to make a monolayer. The smear was allowed to dry out in the air prior to the AFM measurements. All the AFM images and measurements were obtained by an AFM (Multimode, Bruker, USA) operating in tapping mode. Silicon probe with aluminum reflective coating on its back side (TEPSA, Bruker, USA) was employed in AFM imaging. The probe has a spring constant of 20–80 N/m, tip curvature radius of 8 nm and a resonant frequency of 342–394 KHz. In order to direct AFM probe to the desired cells, we used a top-view optical microscope. To analyze the alteration of cell membrane, 5–7 cells at different area from each smear were randomly selected and scanned. In addition, the experiments were repeated several times to rule out artefacts. The AFM images and roughness measurements were processed using the NanoScope Analysis 1.3 (Bruker, USA).

### Statistical analysis

In order to show the distinct difference between the spectral parameters of two groups, namely normal controls and smokers, canonical discriminant function statistics was done using SSPC Statistics Software.

## Conclusion

The findings of this study indicate that the toxins inhaled by smoking produce nano ruptures, nano hemolysis, and morphological deformities of the erythrocytes. The enhanced level of hemolytic metabolites, as shown by spectral analysis for the first time, is well correlated by the observations of atomic force microscopy. The results of this study at least partially explain the causes for cardiovascular diseases and erectile dysfunction associated with excessive smoking.

## Additional Information

**How to cite this article**: Masilamani, V. *et al.* Smoking Induced Hemolysis: Spectral and microscopic investigations. *Sci. Rep.* **6**, 21095; doi: 10.1038/srep21095 (2016).

## Figures and Tables

**Table 1:**
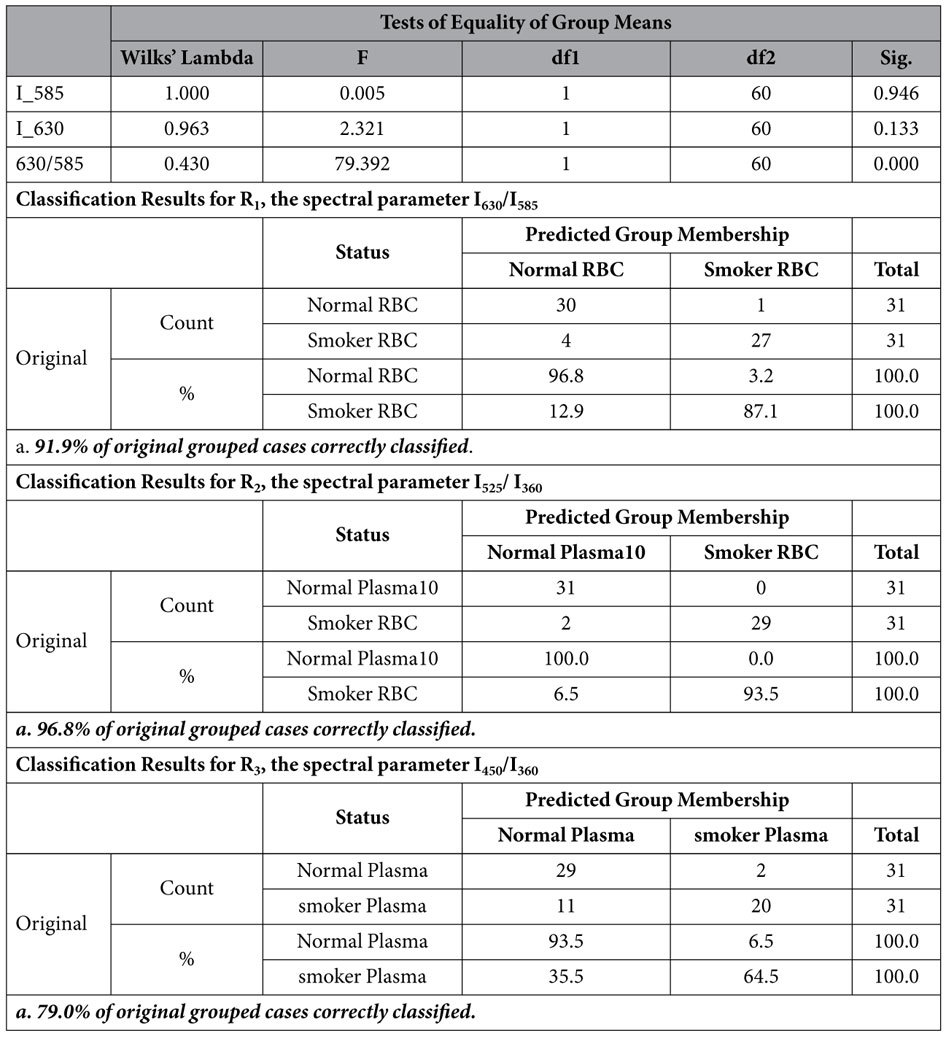
Summary of the canonical discriminant analysis of the spectral data R_1_, R_2_ and R_3_.
